# Maternal obesity programs reduced leptin signaling in the pituitary and altered GH/IGF1 axis function leading to increased adiposity in adult sheep offspring

**DOI:** 10.1371/journal.pone.0181795

**Published:** 2017-08-03

**Authors:** Nuermaimaiti Tuersunjiang, John F. Odhiambo, Desiree R. Shasa, Ashley M. Smith, Peter W. Nathanielsz, Stephen P. Ford

**Affiliations:** Center for the Study of Fetal Programming, Department of Animal Science, University of Wyoming, Laramie, Wyoming, United States of America; University of Cordoba, SPAIN

## Abstract

Studies in rodents highlight a role for leptin in stimulation of pituitary growth hormone (GH) secretion, with an impact on body composition regulation. We have reported that maternal obesity (MO) during ovine pregnancy results in hyperphagia, glucose-insulin dysregulation, increased adiposity, hypercortisolemia and hyperleptinemia in mature offspring subjected to a bout of *ad libitum* feeding. We hypothesized that MO reduces leptin signaling in the pituitary and down regulates the GH/IGF1 axis and increases circulating cortisol leading to increased adiposity in their adult offspring. Male lambs born to MO (n = 6) or control (CON, n = 6) ewes were fed only to requirements until placed on a 12 week *ad libitum* feeding trial at maturity. The pituitary, hypothalamic arcuate nucleus, and liver were collected at necropsy and mRNA and protein expression determined. Plasma cortisol concentrations were increased (P<0.05) in MO vs. CON offspring at the end of the feeding trial. Further, serum concentrations of IGF1 decreased (P<0.01) and GH tended to decrease (P<0.08) in MO vs. CON offspring. Pituitary mRNA and leptin receptor protein expression were decreased in MO vs. CON offspring in association with decreased GH mRNA expression, and decreased IGF1 mRNA and protein expression in liver. Liver 11β-hydroxysteroid dehydrogenase 1 (11βHSD1) expression was increased (P<0.01) and its cofactor hexose-6-phosphate dehydrogenase tended to increase (P<0.06) in MO vs. CON offspring. 11βHSD2 expression remained unchanged. These data indicate that MO induced an increase in liver conversion of cortisone to cortisol in adult offspring and support a role for leptin signaling in the pituitary in mediating offspring adiposity.

## Introduction

Obesity is a major human health concern. The current global obesity epidemic, together with its associated chronic diseases, represents a significant economic cost [1,2]. In a survey carried out between 2003 and 2006, the US obesity rate in women of child bearing age (20–44 years old) was estimated at 32% [3]. Additionally, there is strong evidence that maternal obesity and overnutrition during human pregnancy is associated with an increased incidence of insulin resistance and obesity later in life [2,4,5]. There is also compelling evidence from animal studies that maternal obesity alters offspring phenotype in a similar manner [6–8].

Leptin is produced and secreted by adipocytes and circulates to the hypothalamus where it binds to leptin receptor (*OB-Rb*), signaling the level of adiposity and suppressing appetite [9–11]. Rodents with *leptin* or *OB-Rb* mutations show several phenotypes including infertility, hyperphagia, obesity, and reduced energy expenditure as well as reduced somatotrope numbers [12,13]. There is now strong evidence that *OB-Rb* is also localized to pituitary somatotropes [14–16] and exerts an important regulatory role on the maintenance of growth hormone (GH) stores in somatotropes [15,17]. Recently, Childs et al. [18] reported that a pituitary specific *OB-Rb* mutation in mice resulted in severe GH deficiency and significantly increased adiposity. Further, an *in vivo* study showed that exogenous leptin infusion in *ob/ob* mice restored GH secretion, but hypothalamic growth hormone releasing hormone (GHRH) mRNA expression was not altered [19]. Collectively, these data highlight the importance of leptin in modulating somatotrope GH secretion and thus regulation of hepatic IGF1 secretion.

The GH/IGF1 axis is an important regulator of growth and metabolism through effects on carbohydrate, fat and protein metabolism [20–22]. IGF1 infusion in healthy humans reduces blood insulin and lipid levels [23], and circulating GH levels are negatively correlated with BMI [19,24,25]. Further, GH deficiency is associated with an increased risk of obesity, insulin resistance and diabetes mellitus [26], and these adverse metabolic profiles and increased adiposity in GH deficient adults are improved by GH treatment [27]. Either GH [28] or IGF1 [23] treatments are effective in reducing hyperphagia, obesity, hyperinsulinemia and hypertension in adult rat offspring that were exposed to sub-optimal nutrition *in utero*.

We have developed and characterized a sheep model of maternal overnutrition/obesity [6,29,30]. In this model newborn lambs born to MO ewes have a markedly increased adiposity compared to lambs born to lean ewes fed only to requirements [29]. Further, when adult offspring of MO and CON ewes are subjected to an *ad libitum* feeding challenge, MO offspring exhibited hyperphagia, leading to increased weight gain, glucose dysregulation and hyperleptinemia when compared to offspring born to CON ewes. The increased weight gain of MO offspring in response to the feeding challenge was predominantly due to increased fat accumulation both viscerally and subcutaneously [6,31]. We hypothesized that MO programs reduced pituitary sensitivity to leptin, down regulating development of the GH/IGF1 axis and leading to an increase in adiposity in adult offspring.

## Materials and methods

### Animals

All animal procedures were approved by the University of Wyoming Animal Care and Use Committee. Blood and tissues utilized in the present study were obtained at necropsy from adult male offspring in a study previously reported by Long et al [31]. Offspring were born to either overfed/obese ewes (MO ewes) who exhibited an 85% increase in body weight when overfed from 60 days before conception through late gestation, or lean ewes fed only to requirements (CON ewes) who gained only 15.8% over the same time period. As reported by Long et al. [31] male lambs born to MO (n = 6) or CON (n = 6) ewes were housed together and fed only to requirements until being placed on an 12 week ad libitum feeding trial at maturity (2.5 years of age). Weights and blood samples were obtained throughout the feeding trial. At necropsy, the animals were euthanized with an overdose of sodium pentobarbital (Beuthanasia-D Special; Schering-Plough Animal Health, Union, NJ). Organs and tissues were removed and weighed. For the present study, the pituitary, the arcuate nucleus of the hypothalamus, and the right lobe of the liver were collected and portions snap frozen in liquid nitrogen and stored at –80°C. Pituitary tissue was also fixed in paraformaldehyde and paraffin embedded for immunohistochemical evaluation.

### Biochemical assays

Plasma IGF1 and ACTH concentrations were determined using an Immulite 1000 immunoassay analyzer with a sensitivity of 9 pg/mL (Siemens Medical Solutions, Malvern, PA, USA). IGF1 measurements were completed in a single assay, with an intra-assay coefficient of variation of 5.5%. All ACTH measurements were completed in a single assay, which resulted in intra-assay coefficient of variation of 5.5%. Plasma cortisol was determined in duplicate as previously described [21], using a commercial cortisol RIA kit with a sensitivity of 0.5 μg/dL (Siemens Healthcare Diagnostics, Deerfield, IL, USA). All cortisol measurements were also completed in a single assay and the intra-assay CV for cortisol was 4.5%. Serum GH was quantified in the laboratory of Dr. Dennis Hallford (New Mexico State University) using a double antibody RIA as described by and Hoefler and Hallford [32], and using primary antisera and purified ovine GH provided by the National Hormone and Peptide Program. Further, GH was determined in a single assay and the within assay coefficient of variation was 6.1%.)

### Immunohistochemistry

Immunostaining of GH and OB-Rb in 5μm pituitary tissue sections were accomplished following the general immunohistochemistry protocols described by Ma et al [30]. Briefly, paraffin embedded sections were deparaffinized and rehydrated by routine methods, and then antigen retrieval was accomplished by boiling the sections in citrate buffer. Sections were then probed with rabbit anti-GH (Geneway, San Diego, CA) and goat anti-OB-Rb (Abbiotec, San Diego, CA) primary antibodies followed by fluorescence-labeled anti-rabbit (green) and anti-goat (red) secondary antibodies. Images were visualized using an Olympus BS50 fluorescence microscope and captured digitally using a Retiga ExiFast camera. Pictures at 40 X magnification were taken using QED Imaging software (Media Cybernetics, Silver Spring, MD).

### RNA extraction and real-time PCR

Pituitary and liver tissues were pulverized in liquid nitrogen. Total RNA was extracted from 50 mg of each sample using Trizol reagent (Invitrogen Corp., Carlsbad, CA) treated with DNase I (QIAGEN Inc. Valencia, CA) and then purified by RNeasy mini column (QIAGEN Inc. Valencia, CA) according to the manufacturer’s corresponding protocols. Two μg of purified RNA was used for cDNA synthesis using Promega ImProm-II^™^ Reverse Transcription System (Promega BioSiences, San Luis Obispo, CA) according to the kit protocol. The genes and the primers in real-time PCR experiments are provided in Table 1. All Real-time PCR reactions were conducted using a Bio-Rad IQ5 Realtime-PCR Reaction System (Bio-Rad Laboratories Inc., Hercules, CA) as previously utilized in our laboratory [30]. Final data was analyzed by the C_t_ method relative to 18s rRNA expression.

**Table 1:** Primer sequences of genes evaluated by qPCR.

Gene*	Sense	Anti-sense
OB-Rb	GAT GAG ATG GTG CCA ACA ACT A	TGG GTT TCT ATT TCC CAT GAT C
Leptin	ATC TCA CAC ACG CAG TCC GT	CCA GCA GGT GGA GAA GGT C
GH	TCC AGA ACA CCC AGG TTG CC	CAT CTT CCA GCT CCC GCA TC
IGF1	GAC AGG AAT CGT GGA TGA GTG	AAC AGG TAA CTC GTG CAG AGC
11βHSD1	GCG CCA GAT CCC TGT CTG AT	AGC CCC ATA CCA CCT TCT TT
11βHSD2	AGC AGG AGA CAT GCC GTT TC	GCA ATG CCA AGG CTG CTT
IGFBP3	CAG AAC TTC TCC TCC GAG TCC	CCA CAC ACC AGC AGA AAC C
GHR	ATG AAC CCA TCT GCA TGT GA	TTC AGT CTT CTC ATC AGG GTC A

*Leptin receptor (OB-Rb), growth hormone (GH), insulin-like growth factor-1 (IGF1), 11β-hydroxysteroid dehydrogenase-1 (11β HSD1), 11β-hydroxysteroid dehydrogenase-2 (11β HSD2), growth hormone receptor (GHR), insulin-like growth factor binding protein-3 (IGFBP3).

### Protein extraction and western blotting

Protein was extracted by homogenizing ~100 mg of pulverized pituitary, arcuate nucleus of the hypothalamus and liver tissues in ice-cold lysis buffer using a polytron homogenizer. Homogenates were then centrifuged and the supernatants mixed with 2 × standard SDS sample loading buffer and then boiled at 95°C for 5 minutes. Approximately, 50 μg of protein extracts were separated in SDS-PAGE gels and transferred to nitrocellulose membranes for immunoblotting with primary antibodies for leptin, OB-Rb, GH releasing hormone (GHRH), GH inhibiting hormone (GHIH, somatostatin), GH receptor (GHR), IGF1, IGF-binding protein 3 (IGFBP3), IGF1 receptor(IGF1R), 11 β hydroxysteroid dehydrogenase (HSD)1, 11-β HSD2, Hexose-6-phosphate dehydrogenase (H6PD) and β-actin. The membranes were incubated with appropriate secondary antibodies and then visualized using enhanced chemiluminescence (Amersham Biosciences) and exposure to an X-ray film. The target protein band density was analyzed by ImageQuant TL software (Amersham Biosciences) and normalized according to the density of β-actin as previously described [30].

### Statistical analysis

Repeated variables were analyzed using PROC MIXED procedures of SAS (SAS Institute Inc., Cary, NC) with repeated measures including leptin, ACTH and cortisol analysis from the different time points. The model contained time, treatment and their interaction for repeated measurements. PROC CORR was used for correlation analysis for ACTH and cortisol. Differences in non-repeated variables were determined using analysis of variance by PROC GLM in SAS with treatment in the model statement. Differences were considered significant at *P*≤0.05, tendencies at *P*≤0.10. Data are presented as mean ± SEM.

## Results

### Plasma ACTH and cortisol and liver expression of enzymes that regulate cortisol metabolism

**Fig 1:**
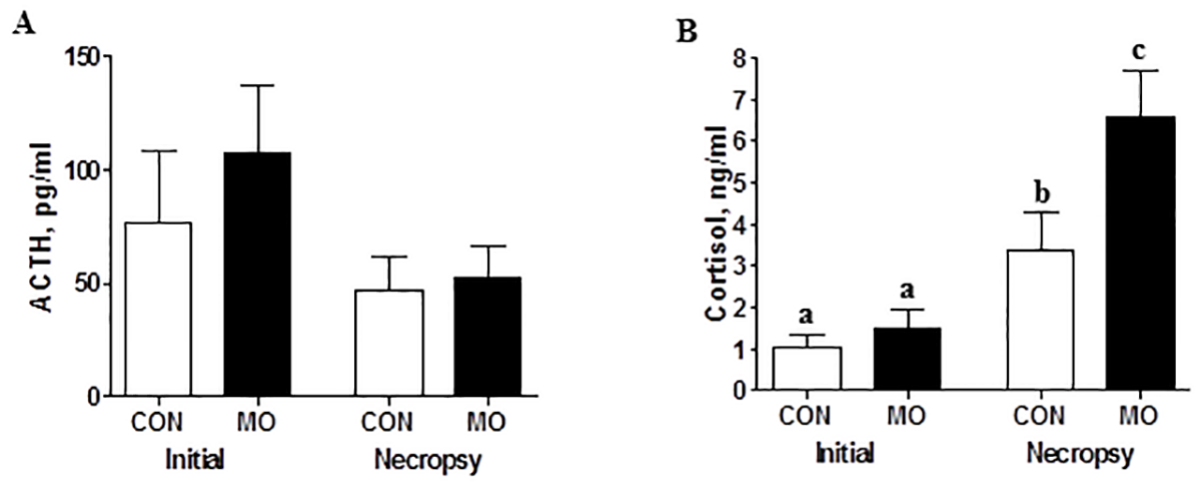
Plasma ACTH (A) and cortisol (B) levels of CON (open bars; n = 6) and MO (solid bars; n = 6) adult male F1 offspring at the initiation and the end of the feeding trial. ^a,b,c^Means ± SEM with different superscripts differ, (*P* < 0.05).

Plasma concentrations of ACTH were similar between the CON and MO offspring both at the beginning and the end of the feeding trial (Fig 1A). However, there was a marked increase (*P* < 0.05) in plasma cortisol concentration in both CON and MO offspring at necropsy compared to the concentrations at initiation of the feeding trial (Fig 1B). Further, while cortisol concentrations were similar between CON and MO offspring at the initiation of the feeding trial, cortisol concentrations were markedly higher in MO than CON offspring at the end of the trial. A significant correlation (*P*<0.01) between ACTH and cortisol levels was found at the initiation of the feeding trial, but no significant correlation (*P*>0.10) was found at necropsy.

**Fig 2:**
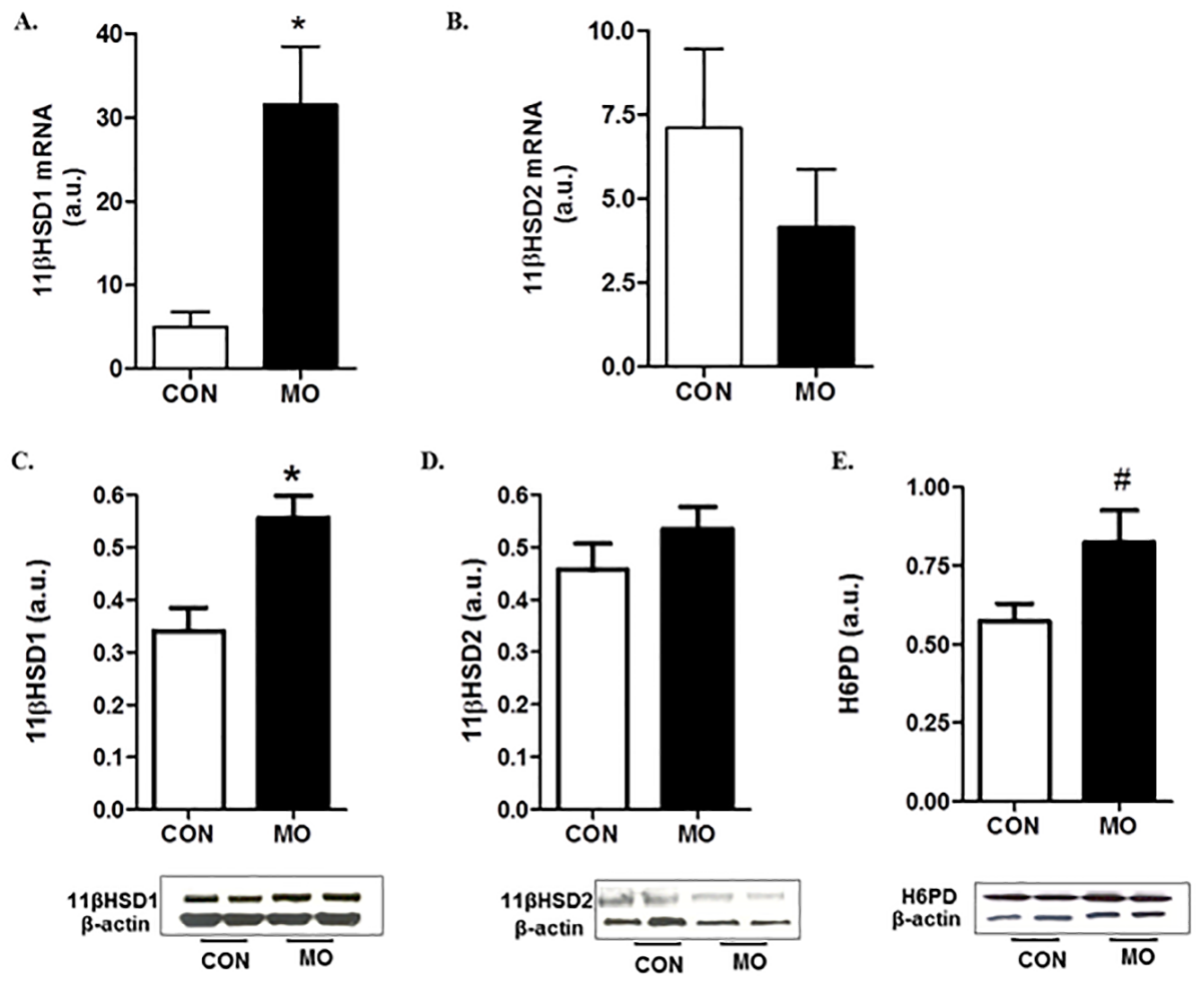
Gene and protein expression of 11βHSD1 (A,C), 11βHSD2 (B,D) and protein expression of H6PD (E) in the livers of CON (open bars, n = 6) and MO (solid bars; n = 6) adult male F1 offspring. ^#^Means ± SEM tended to differ, (*P* < 0.06). *Means ± SEM differ, (*P* < 0.01).

Expression of 11βHSD1, its co-factor H6PD and 11βHSD2 in the livers of adult male offspring was determined to evaluate the potential contribution of peripheral tissues to cortisol production. Expression of 11βHSD1 mRNA and protein was increased (*P*<0.01) and H6PD protein tended to increase (*P*<0.06) in MO vs. CON offspring, but there was no difference in 11βHSD2 mRNA and protein expression between the two groups (Fig 2A–2E).

### Serum GH and IGF1

Serum concentrations of IGF1 were decreased (*P*<0.01) in MO compared to CON offspring at necropsy (327.0 ± 31.0 vs. 443.0 ± 28.0 ng/mL, respectively). There was a tendency for reduced serum concentrations of GH (*P*<0.08) in MO compared with CON offspring at the end of the feeding trial (6.80 ± 0.52 vs. 9.44 ± 0.68 ng/mL, respectively).

### Co-localization of GH and OB-Rb in the pituitary somatotropes

**Fig 3:**
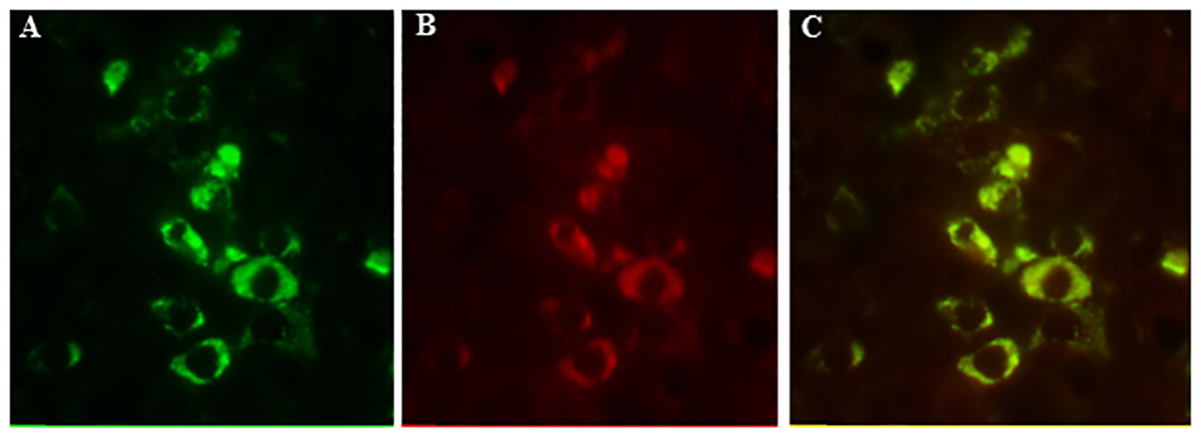
Localization of GH and leptin receptor (OB-Rb) on paraffin embedded pituitary sections using double-labeled immunofluorescence microscopy. GH (green) positive cells (A); OB-Rb (red) positive cells (B); and merged images for GH and OB-Rb expressing cells (C) showing co-localization in somatotropes.

Both GH and OB-Rb were extensively expressed in the ovine pituitary (Fig 3A & 3B) with many somatotropes co-expressing GH and OB-Rb (Fig 3C**)**.

### Expression of the components and the regulators of the GH/IGF1 axis

**Fig 4:**
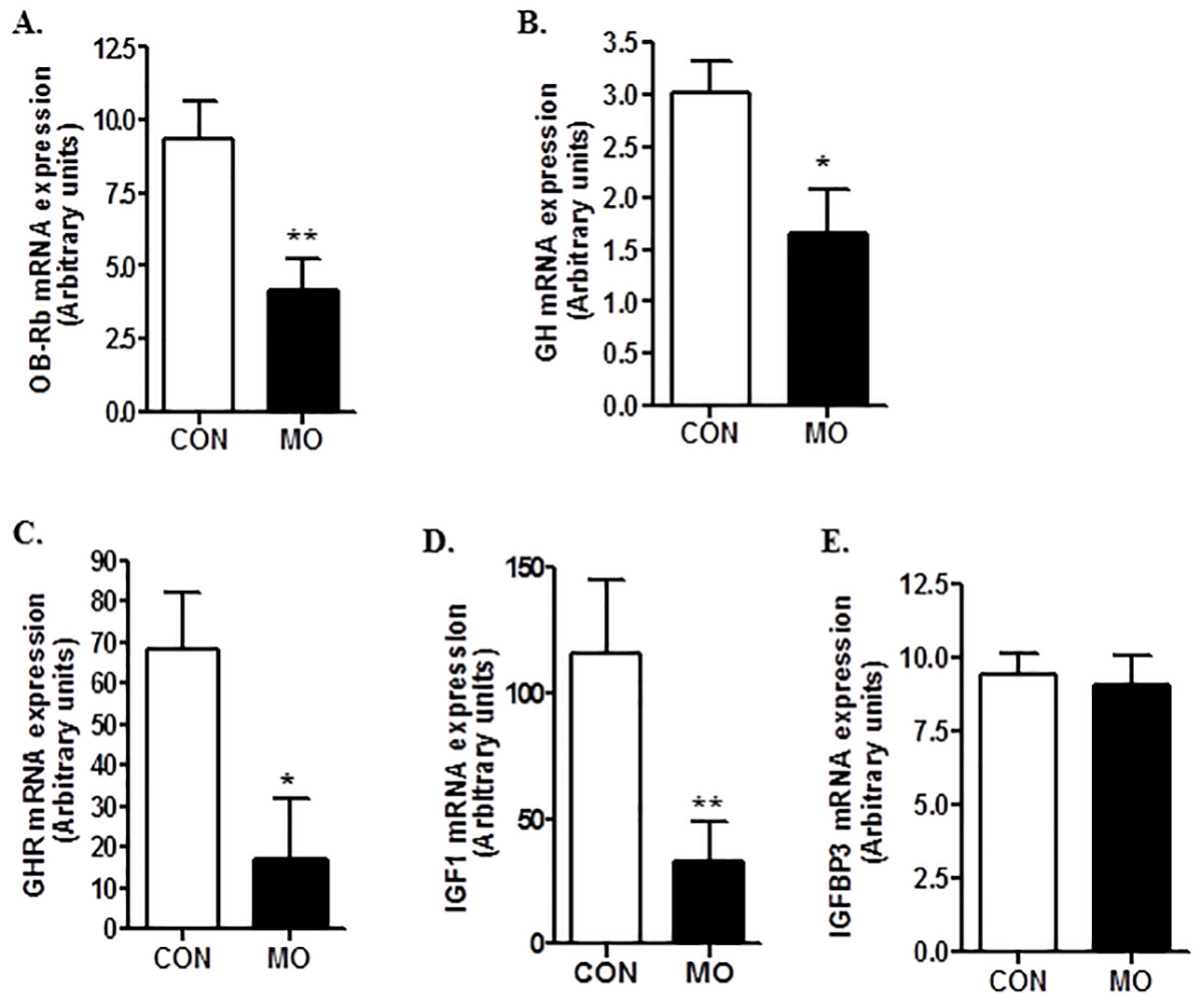
Gene expression of OB-Rb (A), GH (B) in pituitary, and GH receptor (GHR, C), IGF1(D), and IGF binding protein 3 (IGFBP3, E) in liver samples of CON (open bars, n = 6) and MO (solid bars; n = 6) adult male F1 offspring. *Means ± SEM differ, (*P* < 0.05). **Means ± SEM differ, (*P* < 0.01).

**Fig 5:**
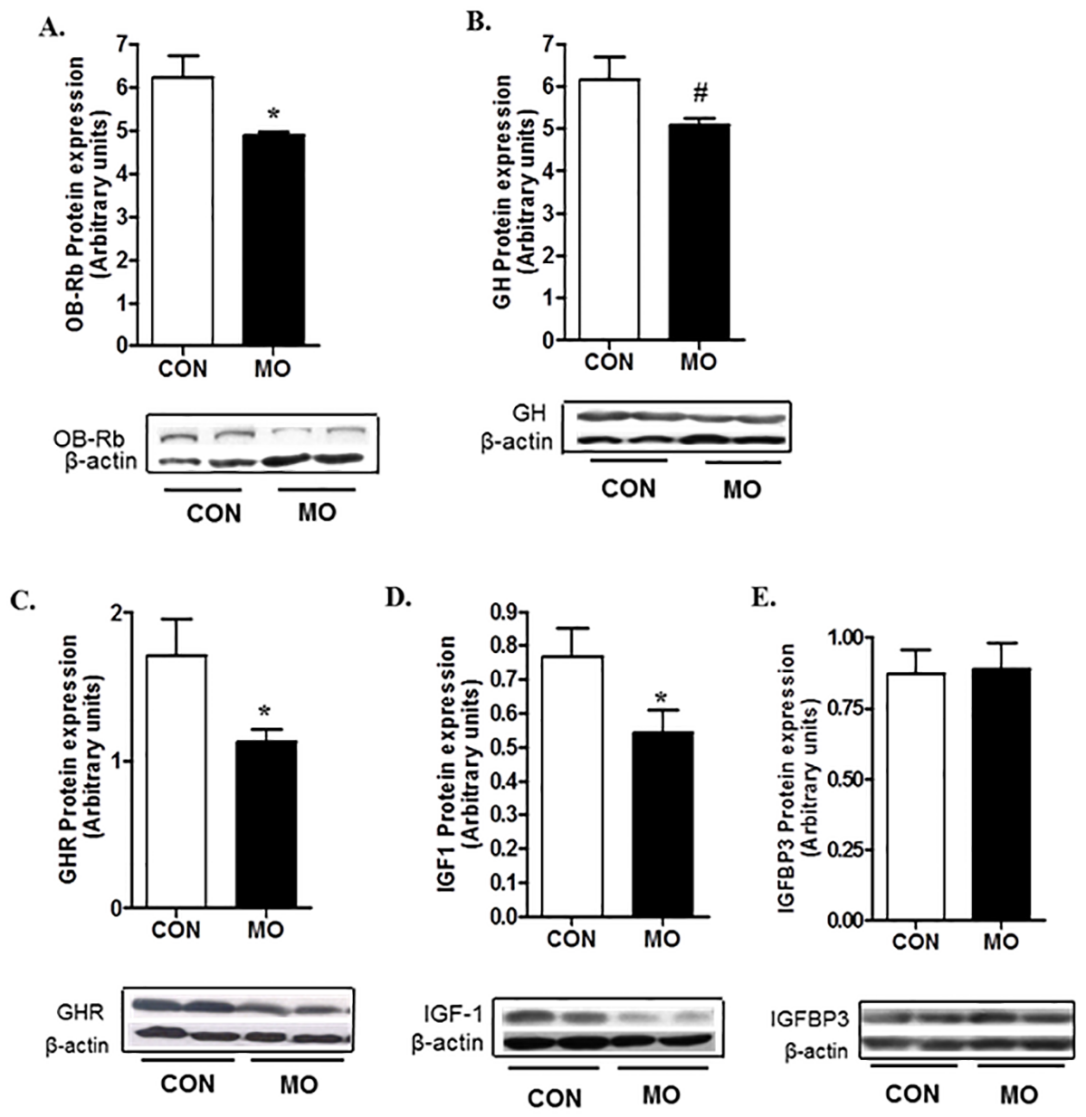
Protein expression of OB-Rb (A), and GH (B) in pituitary, and GHR (C), IGF1 (D), and IGFBP3 (E) in liver samples of CON (open bars, n = 6) and MO (solid bars; n = 6) adult male F1 offspring. *Means ± SEM differ, (*P* < 0.05). ^#^Means ± SEM tended to differ, (*P* < 0. 10).

Protein expression of hypothalamic GHRH and GHIH peptides were determined, and there were no significant differences (*P*>0.10) found in protein expression in either of these peptides in CON and MO adult offspring. Further, the ratio of GHRH/GHIH did not differ between the MO and CON groups (data not shown). However, both mRNA and protein expression of OB-Rb in the pituitary were decreased (*P*<0.05) in MO adults compared with CON adults (Figs 4A & 5A). Pituitary GH mRNA expression was decreased (*P*<0.05) in MO offspring (Fig 4B) but there was only a trend for a decrease (*P*<0.10) in GH protein expression in MO offspring compared with CON offspring (Fig 5B). Further, liver GHR mRNA and protein expression were lower (*P*<0.05) in MO offspring than in CON offspring (Figs 4C & 5C). Liver IGF1 mRNA and protein expression were also lower in MO offspring compared to CON offspring (Figs 4D & 5D). However, no significant differences (*P*>0.05) were found in liver IGFBP3 mRNA or protein expression (Figs 4E & 5E).

## Discussion

It is well-documented that maternal overnutrition/obesity during pregnancy can increase the risk of insulin resistance and obesity in offspring postnatally [33–35]. Further, there is strong evidence that production of leptin, a regulatory hormone mainly produced by adipocytes, can be altered by the maternal nutritional environment and is involved in prenatal or early postnatal programming of increased offspring appetite and obesity [36–38]. The data presented in this study support a novel hypothesis that leptin signaling in the pituitary plays a role in programming of increased adult offspring adiposity by maternal overnutrition/obesity. We have previously shown that lambs born to MO ewes had similar body weights to lambs of CON mothers, but they had an increased percentage of body fat at birth when compared to CON lambs [29]. Further, we reported that the neonatal leptin peak was eliminated by maternal overnutrition/obesity in MO lambs [37,39]. The neonatal leptin peak is present across species, and we have demonstrated that the neonatal leptin peak demonstrated in altricial species is also present in the sheep, a precocial species [37,38,40], and evidence supports the concept that this surge of leptin during the early neonatal period is responsible for development of normal hypothalamic neuronal trajectories that regulate appetite [9,41]. As predicted, the absence of a leptin peak in these MO offspring during the neonatal period [37,39] predisposed them to become hyperphagic in adulthood when they were subjected to an *ad libitum* feeding trial despite having elevated circulating leptin concentration.

Further, we have previously demonstrated [6,31] that male offspring born to MO mothers gain more weight and accumulate more fat when subjected to a postnatal *ad libitum* feeding challenge at maturity than offspring from mothers fed only to requirements. In that study, plasma leptin concentrations were elevated in MO offspring compared with CON offspring, yet MO offspring had a greater feed intake, suggesting that the MO male offspring were leptin resistant. Nevertheless, increased appetite alone cannot explain why the MO adult offspring only increased fat mass rather than lean tissue mass. Rodents with mutations in leptin receptor have low numbers of somatotropes [42,43]. Further, exogenous leptin injection to ob/ob mice restores GH secretion [44]. Recently, Childs et al. [18] demonstrated that a pituitary specific deletion of OB-Rb in mouse caused obesity and dramatic decreases in both somatotrope numbers and GH secretion. The group later reported more extreme cases of GH deficiency and abdominal obesity in male mice that had selective ablation of exon 1 of OB-Rb in somatotropes [45]. This excision of OB-Rb exon 1 resulted in the loss of all isoforms of the leptin receptor and uncovered a broader role for somatotropes as metabolic sensors including sex-specific responses to leptin [45,46].

In our study, we report reduced pituitary OB-Rb expression in MO offspring in addition to co-localization of GH and OB-Rb in the same population of anterior pituitary cells demonstrating a probable functional relationship. This decrease in OB-Rb expression in somatotropes could compromise the function of the GH/IGF1 axis, leading to increased adiposity due to a reduced lipolytic action of GH and IGF1 [18,47,48]. We found that the mRNA expression of both pituitary GH and liver IGF1 were reduced in MO offspring compared to CON offspring. Further, there was a trend towards a decrease in protein expression of GH in the pituitary, and a trend for a decrease in serum GH levels in MO vs. CON offspring. The serum IGF1 concentration was significantly lower in MO offspring than CON offspring, consistent with a decrease in liver IGF1 secretion. It is well established that the hypothalamus has an important role in regulation of the somatotrope axis through GH releasing hormone (GHRH) and somatostatin (GH inhibiting hormone, GHIH) secretion from the arcuate nucleus [49], and that the ratio of these two antagonistic peptides modulate growth hormone secretion [50]. However, as we found no differences in hypothalamic expression of these two peptide hormones, differences in hypothalamic function may not be the driving force for the difference observed in GH and IGF1 levels of MO and CON offspring. Collectively, these results are consistent with the concept that the GH/IGF1 axis in MO offspring was down-regulated in association with reduced leptin signaling in the pituitary.

The GH/ IGF1 axis is one of the most important regulators of growth and metabolism in mammals, due to its regulation of carbohydrate, fat and protein metabolism [51]. With prolonged exposure to GH, protein anabolism, organ growth and lean body mass increases and body fat mass decreases [52,53]. The level of visceral obesity is strongly and negatively associated with both circulating GH and IGF1 levels, and treatment of GH deficient young adults with GH and IGF1 reduces the percentage fat mass and increases the rate of protein synthesis, which contributes to elevated lean muscle mass [54]. GH treatment reduces abdominal obesity in women [55]. Further, greater visceral adiposity and insulin resistance in overweight girls were associated with lower GH and higher cortisol levels [56]. These data indicate that lower GH and/or IGF1 levels are associated with increased visceral adiposity and insulin resistance. The lack of hepatic expression differences in IGFBP-3 in MO vs. CON offspring suggests no role for IGFBP-3 in altering half-life of IGF1 in the blood stream. IGFBP-3 is produced by the liver and released into the systemic circulation, binding to and transporting 70–90% of all circulating IGF [57].

We have reported increased circulating glucose and visceral adiposity as well as reduced pancreatic insulin release in these MO offspring [31], in addition to the lower circulating IGF1 levels and higher cortisol levels at the end of an *ad libitum* feeding trial reported here. The elevated cortisol level was not correlated with ACTH level in MO offspring suggesting either altered adrenocortical responsiveness to ACTH or cortisol production that we and others have shown in the peripheral tissues [58]. Further, Moore et al. [59] showed an inhibitory role for IGF1 on 11βHSD1 activity the key enzyme that converts cortisone to cortisol. Transgenic mice studies show that 11βHSD1 overexpression causes increased visceral adiposity and profound insulin resistance [60]. In contrast, overexpressing 11βHSD2, the enzyme that inactivates cortisol by converting it to cortisone in peripheral tissues protects against obesity and improves insulin resistance [61]. Protein expression of 11βHSD1 and H6PD were increased in MO offspring vs. CON offspring, while no changes were found in 11βHSD2 expression in the liver. Elevation of H6PD expression in the liver could enhance intracellular NADPH availability that drives 11βHSD1 reductase activity resulting in increased local cortisol production from the conversion of cortisone to cortisol. We suggest that the reduced IGF1 level in MO offspring in this study is associated with enhanced 11βHSD1 activity, thus stimulating increased cortisol production. Taken together, the reduction in IGF1 and increased cortisol levels may explain the elevated adiposity in MO offspring. In conclusion, the data presented here provide evidence for the first time that leptin signaling in the pituitary plays a role in programming of adiposity in offspring born to overnourished/obese mothers.

## Supporting information

S1 Table (PDF)
